# Post-exercise serum hepcidin levels were unaffected by hypoxic exposure during prolonged exercise sessions

**DOI:** 10.1371/journal.pone.0183629

**Published:** 2017-08-22

**Authors:** Kazushige Goto, Daichi Sumi, Chihiro Kojima, Aya Ishibashi

**Affiliations:** 1 Graduate School of Sports and Health Science, Ritsumeikan University, Kusatsu, Shiga, Japan; 2 Faculty of Sports and Health Science, Ritsumeikan University, Kusatsu, Shiga, Japan; 3 Japan Institute of Sports Sciences, Kitaku, Tokyo, Japan; Sao Paulo State University, BRAZIL

## Abstract

The purpose of the present study was to determine the influence of hypoxic exposure during prolonged endurance exercise sessions (79 min in total) on post-exercise hepcidin levels in trained male endurance athletes. Ten endurance athletes (mean ± standard deviation; height: 169.8 ± 7.1 cm, weight: 57.1 ± 5.0 kg) conducted two endurance exercise sessions under either a normobaric hypoxic condition [inspired O_2_ fraction (FiO_2_) = 14.5%] or a normoxic condition (FiO_2_ = 20.9%). Exercise consisted of 10 × 3 min running on a treadmill at 95% of maximal oxygen uptake ($\dot{V}O_{2\max}$) with 60s of active rest at 60% of $\dot{V}O_{2\max}$. After 10 min of rest, they subsequently performed 30 min of continuous running at 85% of $\dot{V}O_{2\max}$. Running velocities were significantly lower in the HYPO than in the NOR (*P* < 0.0001). Exercise-induced blood lactate elevation was significantly greater in the HYPO (*P* < 0.01). There were significant increases in plasma interleukin-6, serum iron, and blood glucose levels after exercise, with no significant difference between the trials [interaction (trial × time) or main effect for trial, *P* > 0.05]. Serum hepcidin levels increased significantly 120 min after exercise (HYPO: from 10.7 ± 9.4 ng/mL to 15.8 ± 11.2 ng/mL; NOR: from 7.9 ± 4.7 ng/mL to 13.2 ± 7.9 ng/mL, *P* < 0.05), and no difference was observed between the trials. In conclusion, endurance exercise at lower running velocity in hypoxic conditions resulted in similar post-exercise hepcidin elevations as higher running velocity in normoxic conditions.

## Introduction

Iron deficiency is a frequent diagnosis among athletes, particularly endurance athletes[1–4]. The prevalence of iron deficiency in females competing in various sports was reported to range from 25% to 36%[5,6]. Exercise-induced iron deficiency has been attributed to several factors, including sweating, hemolysis mainly due to heel strike, hematuria, and gastrointestinal bleeding[3,7–9]. Moreover, attention to the influence of hepcidin (a liver-derived, iron-regulating hormone) on iron metabolism is growing[10–17]. Hepcidin regulates iron metabolism [18] by degrading ferroportin (iron export protein) transport channels in the intestine and on the surfaces of macrophages[19,20]. This process decreases dietary iron absorption and iron release from macrophages, which recycle iron from damaged erythrocytes, and eventually leads to low iron availability in the blood[21,22].

Several previous studies have demonstrated that exercise transiently increases hepcidin levels in serum [23–25] and urine[10], which peaks about 3h post-exercise. Furthermore, a cumulative effect of exercise training on elevated hepcidin levels has also been confirmed [13,15,26], although some studies did not find influence of long-term training on hepcidin levels [16,27,28]. Exercise-induced hepcidin elevation is upregulated by the production of inflammatory cytokine interleukin-6 (IL-6)[20], whereas hepcidin response is attenuated by erythropoiesis [28–30], with concomitant elevated erythropoietin (EPO) [29–31]. Since hypoxia is a strong stimulus for promoting erythropoiesis[32], exposure to hypoxia during exercise may lower the magnitude of exercise-induced hepcidin elevation, which is generally observed around 3 h following exercise. Hepcidin levels are suppressed within 48h of continuous exposure to altitude[33]. Badenhorst et al.[34]revealed that following two 8 × 3 min interval training sessions at 85% of maximal oxygen uptake ($\dot{V}O_{2\max}$), hypoxic exposure (F_i_O_2_: 15.1%) for 3h post-exercise successfully attenuated the hepcidin response. In contrast, hypoxic exposure (F_i_O_2_: 14.5%) during endurance exercise sessions did not affect the post-exercise elevation of hepcidin[35]. However, since the exercise protocol in the above study consisted of 5 × 4 min running sets (at 90% of $\dot{V}O_{2\max}$), exposure to hypoxia was shorter than commonly prescribed for daily training among well-trained endurance athletes[36]. Furthermore, the benefit of high-intensity interval training (HIT) in hypoxia has been recently evident for improving endurance capacity[37]. Therefore, impact of hypoxia during exercise regimens consisting of HIT session and continuous exercise session needs to be currently clarified.

Therefore, the present study was designed to compare the post-exercise hepcidin response between prolonged endurance exercise sessions (79 min in total) under normoxic (F_i_O_2_: 20.9%) and hypoxic conditions (F_i_O_2_: 14.5%) in trained male endurance athletes. We hypothesized that the exposure to moderate hypoxia during endurance exercise sessions would attenuate post-exercise hepcidin elevation.

## Methods

### Subjects

Ten male endurance athletes participated in the present study. Their age, height, and body weight [mean ± standard deviation (SD)] were 19.8 ± 0.9 years (19–21 years), 169.8 ± 7.1 cm, and 57.1 ± 5.0 kg, respectively. The average $\dot{V}O_{2\max}$ was 63.4 ± 3.0 mL/kg/min. All athletes were born at and living at sea level, and they had been maintaining specific training (at least more than a year) for long distance running five days per week (approximately 70 km per week). Inclusion criteria were maintenance of training (at least more than a year) and non-smoking. The subjects were informed of the experimental procedures and possible risks involved in this study, and written informed consent was subsequently obtained (August, 2015). The present study was approved by the Ethics Committee for Human Experiments at Ritsumeikan University, Japan. All procedures performed in studies involving human participants were in accordance with the ethical standards of the institutional and/or national research committee and with the 1964 Helsinki declaration and its later amendments or comparable ethical standards.

### Experimental overview

The subjects visited the laboratory four times throughout the experimental period. During the first two visits, two bouts of $\dot{V}O_{2\max}$ testing were completed using a treadmill (Valiant, Load Co., Netherlands) under a normoxic (F_i_O_2_ = 20.9%) or a normobaric hypoxic condition (F_i_O_2_ = 14.5%, equivalent to a simulated altitude of 3,000 m), respectively. These tests were performed at least three days apart. For the determinations of $\dot{V}O_{2\max}$ under hypoxic (F_i_O_2_: 14.5%) and normoxic conditions (F_i_O_2_: 20.9%), identical protocol was used based on preliminary experiment. In brief, the initial running velocity was set at 12 km/h (3.33 m/s), and the running velocity was increased 2 km/h (0.56 m/s) every 2 min to 16 km/h (4.43 m/s). Once the running velocity reached 16 km/h (4.43 m/s), it was further increased 0.6 km/h (0.17 m/s) every 1 min until exhaustion. The first criterion for exhaustion was maintenance of prescribed running velocity. In addition, we have confirmed all subjects met at least two of four criteria ($\dot{V}O_{2}$ plateau, respiratory exchange ratio >1.10, HR of at least 90% of theoretical maximum, and rating of perceived exertion > 9 (10 scale) before determination of exhaustion. During the test, respiratory samples were collected continuously using an automatic gas analyzer (AE300S, Minato Medical Science Co., Ltd, Tokyo, Japan). The data collected were averaged every 30s. The highest value was defined as $\dot{V}O_{2\max}$. Heart rate (HR) was measured throughout the test using a wireless HR monitor (Acculex Plus; Polar Electro Oy, Kempele, Finland). In addition, the subjects indicated their rating of perceived exertion at the end of each stage using 10 scale[38]. The order of the two repeated bouts of $\dot{V}O_{2\max}$ testing under normoxic and hypoxia was randomized.

During the third and fourth sessions, subjects conducted experimental trials in either a normobaric hypoxic (F_i_O_2_ = 14.5%, HYPO) or a normoxic condition (F_i_O_2_, = 20.9%, NOR). Each trial was separated by 1 week. All trials were completed in an environmental chamber, and the normobaric hypoxic condition was produced by insufflating nitrogen into the entire room. Oxygen and carbon dioxide concentrations within a chamber were continuously monitored. The temperature and relative humidity in the environmental chamber was maintained at 22°C and 50%, respectively.

The two trials were performed in a randomized crossover design (with at least a week interval between the two trials), and the subjects were not informed of whether the trial was conducted under normoxic or hypoxic conditions. The HYPO and NOR trials were started after at least a week following $\dot{V}O_{2\max}$ test. All exercise sessions, including 5 min of warm-up at 60% of $\dot{V}O_{2\max}$ (using $\dot{V}O_{2\max}$ evaluated under hypoxic or normoxic conditions), were conducted under hypoxic condition in the HYPO or normoxic condition in the NOR. After warm-up exercise, the subjects initially performed HIT consisting of 10 × 3 min running at 95% of $\dot{V}O_{2\max}$ with 60s of active rest at 60% of $\dot{V}O_{2\max}$. After 10 min of rest, they subsequently started 30 min of continuous running at 85% of $\dot{V}O_{2\max}$. The total duration for exercise sessions was 79 min. We have selected the HIT protocol, because there are several evidences to support the efficacy of HIT on endurance capacity[37,39]. Furthermore, 30 min of continuous exercise at submaximal intensity (85% of $\dot{V}O_{2\max}$) was also added to ensure sufficient duration of the exercise. Since running velocities during HIT and continuous running were determined based on the $\dot{V}O_{2\max}$ evaluated under normoxic or hypoxic conditions, selected velocities were different between the two trials. The subjects ran at lower velocity during HIT in the HYPO [16.2 ± 0.9 km/h (4.49 ± 0.25 m/s)] than in the NOR [18.0 ± 0.8 km/h (4.99 ± 0.24 m/s), *P* < 0.0001]. During the continuous exercise session, the subjects also ran at lower velocity in the HYPO [14.5 ± 0.8 km/h (4.02 ± 0.23 m/s)] than in the NOR [16.1 ± 0.7 km/h (4.47 ± 0.21 m/s), *P* < 0.0001].

Venous blood samples were collected before exercise, 0, 60, and 120 min after completing exercise to monitor time-course changes in serum hepcidin levels.

All subjects were asked to maintain physical activity levels during experimental period.

### Measurements

#### Physiological variables during exercise sessions

Percutaneous oxygen saturation (SpO_2_) was evaluated at sets 1, 5, and 10 during HIT and every 10 min during the continuous exercise session. The data at each point were collected during 15 s using a finger pulse oximeter (Smart Pulse, Fukuoka Denshi, Tokyo, Japan). Heart rate (HR) was continuously recorded during exercise every 5 s using a wireless HR monitor (Acculex Plus; Polar Electro Oy, Kempele, Finland).

### Blood variables

Following an overnight fast, the subjects visited the test site laboratory at 7:30 in the morning and rested before the first blood collection. A baseline blood sample was subsequently obtained. Blood samples were also collected at 0, 60, 120 min after exercise sessions. Serum and plasma samples were obtained after a 10 min centrifugation at 4°C, and the samples were stored at -80°C until analysis. From the obtained blood samples, blood glucose, lactate, serum iron, hepcidin, and plasma IL-6 levels were measured. Blood glucose and lactate levels were measured using a glucose analyzer (Free style, Nipro Co., Osaka, Japan) and a lactate analyzer (Lactate Pro, Arkray Co., Kyoto, Japan) immediately after blood collection. Blood glucose level measurements were duplicated, and average values were used for analysis. Serum iron levels were measured at a clinical laboratory (SRL Inc., Tokyo, Japan). The intra-assay coefficient of variance (CV) for serum iron measurement was 1.3%. Plasma IL-6 levels were measured with enzyme-linked immunosorbent assay (ELISA) kits (Human IL-6 Quantikine HS ELISA kit, R&D Systems, Minneapolis, USA). The sample was analyzed in duplicate, and average values were determined. The intra-assay CV was 4.4%. Serum hepcidin levels were analyzed with an ELISA kit (R&D Systems, Minneapolis, MN, USA), and the intra-assay CV was 2.3%.

### Statistical analysis

All data are presented as means ± SD. A two-way analysis of variance (ANOVA) with repeated measures was used to confirm the interaction (trial × time). When the ANOVA revealed a significant interaction or main effect, a Tukey-Kramer test was performed as a post hoc analysis to identify differences. The areas under the curves (AUC) for plasma IL-6 and serum hepcidin levels were compared with respect to the HYPO and NOR conditions using a paired-test. In addition, Cohen’s d (for paired t-test) or partial eta squared (partial η^2^ for two-way ANOVA with repeated measures) were calculated to quantify effect sizes (ES). For all tests, *P* values < 0.05 were considered to indicate statistical significance.

## Results

### Serum hepcidin

Table 1 presents time course changes in serum hepcidin levels. Serum hepcidin levels for one subject was not able to detect due to below the limit of the detection. Exercise significantly increased serum hepcidin levels (main effect for time: *P* < 0.05, ES = 0.52), and a significant increase from pre-exercise levels was observed 120 min after completing exercise sessions in both trials (*P* < 0.05 for HYPO, *P* < 0.01 for NOR). However, exercise-induced hepcidin responses were similar for the HYPO and the NOR trials [interaction (trial × time, *P* > 0.05, ES = 0.09) or main effect for trials (*P* > 0.05, ES = 0.05), with no significant difference between the trials. The AUC for serum hepcidin levels during exercise and the post-exercise period did not differ significantly (*P* > 0.05, ES = 0.30) between the HYPO (2421 ± 1692 ng/mL) and the NOR (1991 ± 1110 ng/mL).

**Table 1:** Changes in serum hepcidin levels.

		Pre	Post0 min	Post60 min	Post120 min
Hepcidin (ng/mL)	NOR	7.9 ± 4.7	10.1 ± 6.1	9.7 ± 6.0	13.2 ± 7.9*
	HYPO	10.7 ± 9.4	12.7 ± 9.4	11.8 ± 7.6	15.8 ± 11.2*

Values are means ± SD (n = 9).

*: Significant difference vs. Pre.

### Physiological variables during exercise sessions

In the HYPO, SpO_2_ levels were significantly lower during both HIT (average SpO_2_: HYP 78 ± 3% vs. NOR 94 ± 2%, *P* < 0.001, ES = 6.28) and continuous exercise (average SpO_2_: HYP 79 ± 3% vs. NOR 95 ± 2%, *P* < 0.001, ES = 5.88). The HR neither differed significantly between the HYPO and the NOR during HIT (average HR: HYP 178 ± 7 bpm vs. NOR 182 ± 5 bpm, ns, ES = 0.66) nor during continuous exercise (average HR: HYP 174 ± 9 bpm vs. NOR 177 ± 9 bpm, ns, ES = 0.33).

### Blood glucose, lactate, serum iron, and plasma IL-6

Table 2 presents time-course changes in blood glucose, lactate, serum iron, and plasma IL-6 levels. Blood glucose levels did not change significantly immediately after the exercise, but they significantly decreased 60 min and 120 min post-exercise compared with pre-exercise levels in both trials (main effect for time: *P* < 0.05, ES = 15.32). However, there was no significant difference between the two trials at any time points (interaction: *P* > 0.05, ES = 0.10, main effect for trial: *P* > 0.05, ES = 0.01). Blood lactate levels significantly increased immediately after the exercise compared with pre-exercise levels only in the HYPO (*P* < 0.001, ES = 0.67), with a significant difference from that in the NOR (*P* < 0.01, ES = 0.51). Both trials showed similar increases in serum iron levels compared with pre-exercise levels during the post-exercise period (main effect for time: *P* < 0.01, ES = 0.91), with no difference between the HYPO and NOR at any time points (interaction: *P* > 0.05, ES = 0.30, main effect for trial: *P* > 0.05, ES = 0.11). The HYPO and NOR trials showed similar increases in plasma IL-6 levels (main effect for time: *P* < 0.001, ES = 0.86), with no significant difference between the HYPO and NOR at any time points (interaction: *P* > 0.05, ES = 0.13, main effect for trial: *P* > 0.05, ES = 0.04). Furthermore, the AUC for plasma IL-6 levels during exercise and the post-exercise period was not significantly different (*P* > 0.05, ES = 0.20) between the HYPO (621.8 ± 272.8 pg/mL) and the NOR (674.5 ± 264.2 pg/mL).

**Table 2:** Changes in blood glucose, lactate, serum iron and plasma IL-6 levels.

		Pre	Post0 min	Post60 min	Post120 min
Glucose (mg/dL)	NOR	91 ± 6	88 ± 10	81 ± 8*	79 ± 7*
	HYPO	92 ± 10	93 ± 11*	78 ± 11*	83 ± 13*
Lactate (mmol/L)	NOR	1.7 ± 0.5	3.2 ± 2.0	1.9 ± 0.8	2.0 ± 0.7
	HYPO	2.0 ± 0.8	5.1 ± 1.6*†	1.9 ± 0.8	2.1 ± 0.7
Iron (μg/dL)	NOR	102 ± 38	146 ± 41*	151 ± 38*	149 ± 34*
	HYPO	94 ± 41	130 ± 48*	127 ± 41*	125 ± 42*
IL-6 (pg/mL)	NOR	0.5 ± 0.2	5.9 ± 2.3*	3.4 ± 1.3*	2.0 ± 1.0*
	HYPO	0.6 ± 0.4	5.2 ± 2.4*	2.9 ± 1.3*	2.0 ± 0.7*

Values are means ± SD (n = 10 for glucose, lactate and iron levels. n = 9 for IL-6 levels).

*: Significant difference vs. Pre.

†: Significant difference vs. NOR.

## Discussion

The main finding of the present study was that endurance exercise at lower running velocity in hypoxic conditions (F_i_O_2_ = 14.5%) resulted in similar post-exercise hepcidin elevations as higher running velocity in normoxic conditions. The finding is consistent with a recently published study that demonstrated a similar exercise-induced hepcidin response to relatively short (about 31 min in total) interval exercise sessions in hypoxic and normoxic conditions[35]. Considering that exercise-induced hepcidin elevation was successfully impaired by hypoxic exposure for 3h post-exercise[34], recovering under hypoxic conditions could contribute to maintaining appropriate iron levels by reducing hepcidin production.

During endurance exercise sessions, SpO_2_ levels remained significantly lower in the HYPO than in the NOR. Since $\dot{V}O_{2\max}$ is expected to be lower under hypoxic conditions[40], we designed the study to match the relative exercise intensity (percentage relative to $\dot{V}O_{2\max}$ evaluated under normoxic or hypoxic conditions) in NOR and HYPO conditions. Consequently, $\dot{V}O_{2\max}$ was significantly lower in the HYPO (44.6 ± 3.8 mL/kg/min) than in the NOR (63.4 ± 3.0 mL/kg/min, *P* < 0.05). Moreover, the running velocities during both HIT and continuous exercise sessions were significantly lower in the HYPO, and the HYPO also showed significantly lower energy expenditure. However, since HR and RPE during exercise sessions did not differ significantly between the HYPO and NOR conditions, the physiological stress appeared to be similar between the trials.

Exercise, particularly running, increases hepcidin levels during exercise[10,24,34,41]. In accordance with previous studies, serum hepcidin levels in the present study were significantly elevated at 120 min after completing exercise sessions in both trials. However, post-exercise hepcidin levels were not significantly different between the two trials. A potential factor for similar levels of post-exercise hepcidin would be the similarity in the exercise-induced IL-6 response. Several physiological factors are involved in hepcidin production in the liver, and the primary mediator for hepcidin production is suggested to be inflammatory cytokine IL-6[20]. Since both trials caused a similar increase in plasma IL-6, the similarity in post-exercise hepcidin levels may be reasonable. This notion is supported by a previous result indicating that IL-6 and hepcidin responses were not affected by hypoxic exposure during 31 min of interval exercise sessions[35]. Aside from IL-6, exercise-induced hemolysis stimulates hepcidin production [10]. Running elicits hemolysis rather than other types of exercise modalities including cycling[42], which is reflected by a greater reduction of serum haptoglobin with lower free hemoglobin levels[10]. Unfortunately, we did not determine changes in these variables to evaluate the magnitude of hemolysis. However, exercise-induced elevation of serum iron, which was suggested to reflect hemolysis[43], was not significantly different between the NOR and HYPO conditions, suggesting that exercise-induced hemolysis was comparable between the two trials. Several previous studies demonstrated that serum hepcidin levels were elevated at 3h after exercise[24], although urinary hepcidin levels revealed peak levels at 6h after 60 min of running[11]. Because we collected a series of blood samples up to 120 min post-exercise, there was a possibility that serum hepcidin levels presented further increases after these time points. However, the exercise sessions in both trials lasted about 80 min in total, and about 3h and 20 min passed from the onset of exercise to the final blood drawing during the post-exercise period. Therefore, it is considered that post-exercise hepcidin levels observed 120 min after exercise sessions would reflect, approximately, peak values.

Several limitations should be carefully considered when interpreting these results. The running velocities during HIT and 30 min of continuous exercise was different between the two trials, because we selected the same relative intensities (% of $\dot{V}O_{2\max}$) in response to $\dot{V}O_{2\max}$ evaluated under normoxic or hypoxic conditions. Although the use of the same relative intensity between HYPO and NOR was in accordance with experimental design among previous studies[35,40,44], lower running velocities in the HYPO may have had influence on the present results. Therefore, further comparison under the same absolute intensities (running velocities) would be meaningful. In addition, we did not prepare a control group who did not exercise, as we attempted to determine the impact of hypoxic exposure during prolonged endurance exercise sessions on post-exercise hepcidin levels. Hepcidin levels display a diurnal rhythm, i.e., lowest in the morning and then gradually increase throughout the day[31]. Thus, post-exercise elevation of serum hepcidin levels may, at least in part, be attributed to diurnal rhythm. In contrast, Peeling et al. [11]reported that 60 min of running significantly increased serum hepcidin levels at 3h post-exercise, but a rest condition without exercise did not show an increase in hepcidin levels at the identical time point. Collectively, the elevation of hepcidin at 120 min after endurance exercise sessions would have been caused by the exercise stimulus.

## Conclusions

Endurance exercise, consisting of HIT and continuous exercise, acutely increased serum hepcidin levels at 120 min during post-exercise period compared with pre-exercise levels. However, the post-exercise levels of plasma IL-6 and serum hepcidin were not significantly different when the exercise sessions were completed under normobaric hypoxic conditions (a simulated altitude of 3000m). Therefore, it appears that endurance exercise at lower running velocity in hypoxic conditions results in similar post-exercise hepcidin elevations as higher running velocity in normoxic conditions.
